# Beyond coverage: why Ayushman Bharat-Pradhan Mantri Jan Arogya Yojana struggles to deliver healthcare in Jammu and Kashmir

**DOI:** 10.3389/fpubh.2026.1783458

**Published:** 2026-03-09

**Authors:** Esarul Ayub, Olusiji Adebola Lasekan, Blessy Sarah Mathew, Margot Teresa Godoy Pena, Abdul Wahid Bhat

**Affiliations:** 1 Lovely Professional University, Phagwara, Punjab, India; 2 Universidad Católica de Temuco, Temuco, Chile; 3 Universidad de La Frontera, Temuco, Chile; 4 Government Degree College, Kishtwar, Jammu and Kashmir, India

**Keywords:** Ayushman Bharat, beneficiary experiences, financial and health literacy and awareness gaps, health scheme utilization, Jammu and Kashmir, PM-JAY, public health policy

## Abstract

Ayushman Bharat-Pradhan Mantri Jan Arogya Yojana (PM-JAY), the biggest publicly financed health insurance program in the world, has substantial use issues, especially in places like Jammu and Kashmir that are affected by conflict. Despite the program’s goal of ensuring equal and cashless access to healthcare, the existing literature does not offer region-specific insights into the actual obstacles preventing its adoption. This study intends to identify and measure the physical, administrative, financial, and informational hurdles experiences by the beneficiaries in the urban and semi-rural areas of Jammu and Kashmir face when utilizing PM-JAY. Primary data was gathered through structured interviews with 320 randomly chosen PM-JAY recipients using a cross-sectional approach. To evaluate the relative effects of various obstacles on scheme use, the study used binary logistic regression, chi-square tests, Pearson correlation, and descriptive statistics. Despite being enrolled, only 48.4% of enrollees reported accessing PM-JAY services. The most significant positive predictors of usage were health literacy and awareness gaps, notably knowledge of empanelled hospital lists (OR = 1.245, *p* < 0.05) and awareness of scheme entitlement (OR = 1.253, *p* < 0.05). A statistically significant negative factor was inadequate road infrastructure (OR = 0.761, *p* < 0.05). Despite being often mentioned, administrative and financial obstacles were surprisingly not significant in the regression model. There were no discernible variations in use between the two districts, indicating problems with systemic access. Increasing PM-JAY use in disadvantaged areas requires more than just financial incentives; it also requires better physical infrastructure and informational availability. To convert coverage into useful access, policymakers should concentrate on region-specific IEC initiatives, transportation connectivity, and health system navigation support mechanisms. These interventions are essential for translating PM-JAY’s theoretical coverage into meaningful, equitable healthcare access, particularly in underserved and fragile regions like Jammu and Kashmir.

## 1 Introduction

Healthcare transformation in India is driven by the huge changes made to attain universal health coverage (UHC) and fill care gaps. The AB-HWCs was renamed as Ayushman Arogya Mandir with the tag -line Arogyam Parmam Dhanam and the focus is to give preventative, promotive, curative, rehabilitative and palliative care keeping in view the sustainable developmental goal (SDG) -3 related to good health and wellbeing. Other initiatives being taken by the government include Ayushman Bharat Pradhan Mantri Jan Arogya Yojana (AB-PMJAY) and Social Endeavour for Health and Telemedicine (SEHAT) aimed at enhancing access to healthcare with the provision of insurance cover and ensuring equitable coverage of basic health services. The plan is one of the biggest publicly funded health assurance plans in the world and is said to guarantee the element of financial security and better access and utilization of the services (1). Although this ambitious structure, the recent evidence indicates that the real program utilization of PM-JAY services will still be restricted because of various structural, informational and contextual disincentives. The research in various Indian contexts shows that out-of-pocket spending, documentation barrier, knowledge deficiency, and inadequate transportation infrastructures still compromise the effectiveness of the scheme (2). It is more of a crisis in such areas as Jammu and Kashmir (J&K) where the healthcare is already complicated due to armed conflict, mountains, and institutional instability (3). This paper pays attention to two distinct districts, Jammu and Baramulla, of the Jammu and Kashmir Union territory, with the objective to evaluate impediments to verified beneficiaries of PM-JAY. Jammu is a relatively urban administrative center as compared to Baramulla which is semi-rural and is always exposed to conflicts and infrastructure shortages. The diversity of region in Jammu and Kashmir offers a critical focal point in evaluating the contextualized barriers mediating scheme access by even households that are qualified to participate in this scheme.

The objectives of this study are;

- To identify the most significant financial, administrative, informational, and physical barriers to PM-JAY utilization;
- To compare how these barriers operate differently across the urban and semi-rural settings of Jammu and Baramulla;

By offering statistically grounded, district-level insights, this research adds to a growing body of evidence on how the design and delivery of public health insurance schemes must evolve to ensure true universality. In doing so, it seeks to inform targeted, evidence-based policy responses for improving healthcare access and financial protection in fragile settings like Jammu and Kashmir (4).

## 2 Review of literature

Universal health coverage (UHC) has been discussed as one of the key foundations of global and domestic health systems, targeting the concept of assuring that everyone can get the health services they require without undergoing a process of deprivation. According to the World Health Organization (5), structural, financial and health literacy and awareness gaps are some bottlenecks that continue to pose a challenge to UHC, particularly in low- and middle-income countries. In India, the Ayushman Bharat PM-JAY program was introduced in order to address these risks, and recent reviews indicate evidence of varied success with regards to alleviating access disparities and out-of-pocket spending. According to Evans et al. (1), UHC needs to be viewed as a developmental prerogative in contrast to a target of the health sector. Nevertheless, research has shown that to date the lack of awareness, informal payments, and even rejection of the claims remain a frequent issue experienced by PM-JAY beneficiaries despite the ambitious architecture of the latter (6). Alebachew et al. (7) also found that there should be a strong monitoring system to monitor UHC progress in Ethiopia, which is relevant regardless of whether the financing is going to be public or not. As it was in the Indian context, Reddy et al. (8) and Garg et.al (2) mention that administrative bottlenecks often weigh down the promise of cashless hospitalization in the form of excessive bureaucracy surrounding the documentation process and inefficient grievance redressal. The same is reflected in Singh and Khokhar (9) who also document the disastrous health spending levels even within enrolled households in urban Delhi. Similarly, Owais et al. (3) report that, in Jammu and Kashmir, the prevalence of out-of-pocket spending remains because of low awareness, low transportation availability, and short-comings in provider network. In the article dealing with the issues of how people think about national health insurance (10) in South Africa, the authors stress that trust and communication are some of the most important elements in the successful implementation of health schemes. Other issues that have been raised include the fact that even though the coverage is growing rapidly under PM-JAY, system inefficiency gaps are not filled (11). A national review by Prinja et al. (33) was able to show an increase in healthcare utilization through various systems of public health insurance; however, the statements were uneven among states in their influence of financial protection. Devadasan et al. (4) and Boerma et al. (12) indicate that granular monitoring is important to realize utilization patterns in the district or regional levels. The available literature, however, is inadequate or unrepresentative in conflict-prone and mountainous regions such as J&K. According to the National Family Health Survey (NFHS-5) (13), (32) and the Press Information Bureau reports (13), extensive variation in healthcare availability in the state is observed, which warrants research of greater depth. Though the earlier studies have grouped the kind of barriers addressed as city specific (financial, administrative, informational, and physical) (14) little has the work done in the nuance of the statistical picture of the independent impact of each barrier type on utilization levels, particularly in conflict or remote regions. Here, this paper fills in that gap by using logistic regression on the primary survey data in J&K and provides a more nuanced picture of which constraints are the most relevant-and among whom.

The Pradhan Mantri Jan Arogya Yojana (PM-JAY) is a flagship public health insurance initiative in India designed to provide financial protection against catastrophic health expenditures for socioeconomically disadvantaged households. The scheme offers coverage of up to INR 5 lakh per family per year and encompasses a wide range of healthcare services, including secondary and tertiary hospitalization, surgeries, diagnostics, and medicines (15, 16). A substantial proportion of the benefit package is oriented toward surgical care, with nearly 65% of listed procedures being surgical in nature, and approximately 82% of empanelled hospitals equipped to provide surgical services (17). High-cost specialties such as cardiology and orthopedics account for a significant share of utilization, while radiodiagnosis and cardiothoracic and vascular surgery (CTVS) represent some of the most expensive service packages under the scheme (34). Service delivery under PM-JAY is contingent upon the empanelment of public and private healthcare facilities, with public sector hospitals constituting more than half of the empanelled network nationwide (18). However, the empanelment process has been uneven, with a higher concentration of participating facilities in states that had prior experience with publicly financed health insurance, resulting in regional disparities in service availability (18). In regions such as Jammu and Kashmir, bureaucratic inefficiencies, infrastructural constraints, and limited private sector participation further complicate provider empanelment and restrict practical access to covered services. Despite the scheme’s extensive benefit design, utilization remains suboptimal, largely due to inadequate beneficiary awareness, weak information dissemination mechanisms, and limited understanding of entitlements (19). Moreover, financial barriers persist in practice, as beneficiaries often incur out-of-pocket expenses due to delayed claim reimbursements, informal charges, and inefficiencies in hospital-based administrative processes, undermining the scheme’s cashless promise (20). These challenges highlight the need for improvements in PM-JAY’s design and implementation to enhance system responsiveness, strengthen provider participation, and reduce residual financial burdens on beneficiaries, particularly in structurally and institutionally constrained regions (21).

Despite a growing body of national-level evidence on PM-JAY and publicly financed health insurance schemes in India, three critical gaps remain. First, existing studies predominantly emphasize financial protection outcomes or aggregate utilization trends, with limited attention to disentangling the independent effects of financial, administrative, informational, and physical barriers within a unified analytical framework. Second, empirical evidence from conflict-affected and geographically constrained regions such as Jammu and Kashmir remains sparse, despite contextual factors such as terrain, infrastructure deficits, mobility restrictions, and institutional fragility potentially reshaping access dynamics. Third, few studies employ multivariate, item-level regression approaches to assess which barriers retain statistical significance when examined simultaneously. Addressing these gaps, the present study applies a barrier-specific logistic regression framework to primary survey data from Jammu and Baramulla districts, thereby generating context-sensitive and statistically disaggregated insights into the determinants of PM-JAY utilization in a fragile setting.

## 3 Conceptual framework

This study is guided by an adapted Andersen’s Behavioral Model of Health Services Use, which explains healthcare utilization as a function of predisposing characteristics, enabling resources, and need operating within a broader health system and environmental context (22, 23). In the PM-JAY context, utilization is not a simple outcome of insurance enrollment; rather, it reflects whether beneficiaries can translate eligibility into actual service use through the combined effects of information, administrative navigability, affordability, and physical accessibility.

To operationalize “access” more explicitly, the framework is complemented by the patient-centred access model proposed by Levesque et al. (24), which conceptualizes access as a matching process between (i) health system/service characteristics (e.g., availability, affordability, acceptability, appropriateness) and (ii) population abilities (e.g., ability to perceive, seek, reach, pay, and engage). This combined framework is particularly suitable for PM-JAY in Jammu and Kashmir because it allows insurance coverage (a financial enabler) to be analysed alongside the practical constraints that often determine whether coverage becomes meaningful utilization, especially in underserved or fragile settings (4, 12, 25).

**Figure 1:**
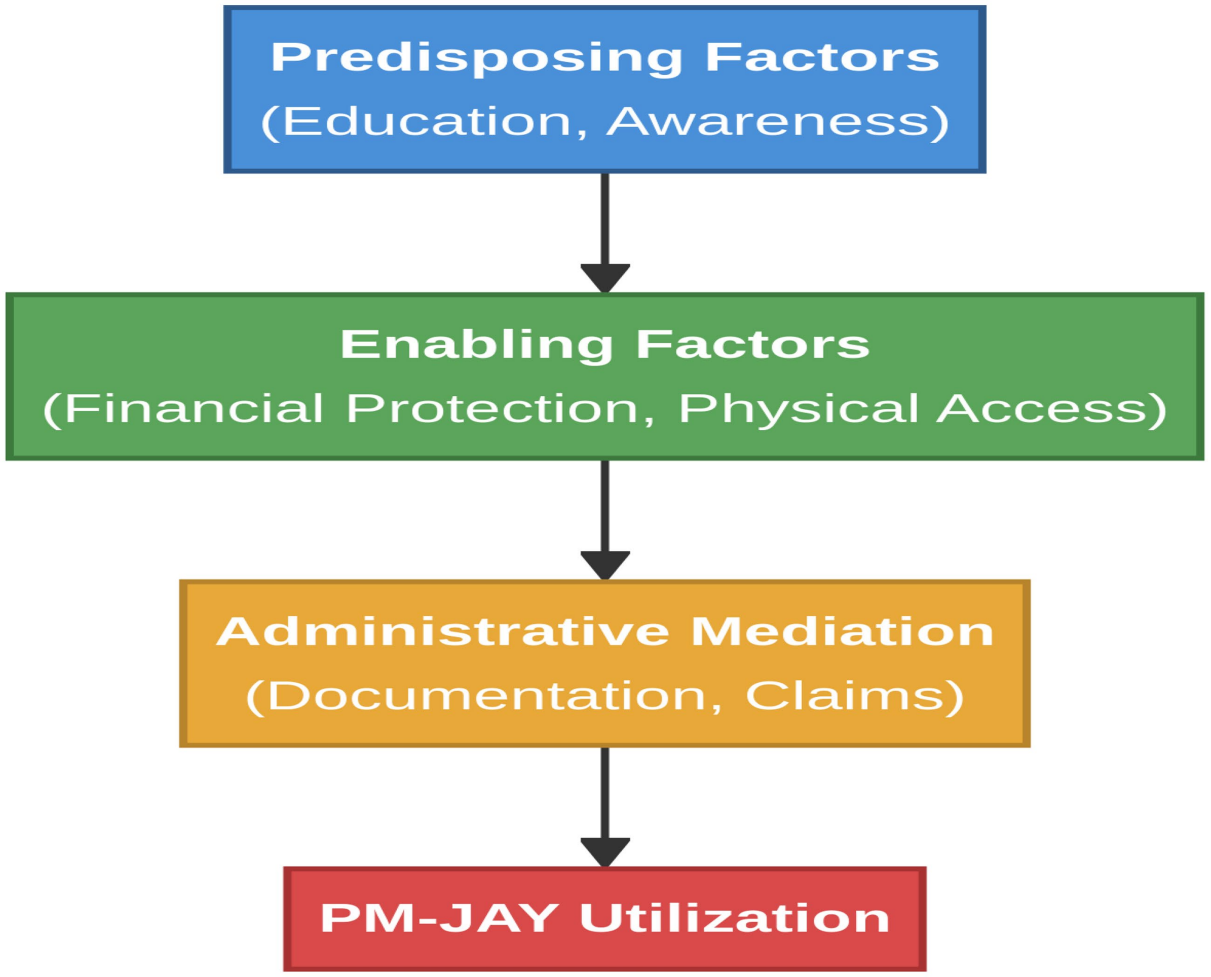
Conceptual framework adapted from Andersen’s behavioral model of health services use illustrating the pathways through which predisposing, enabling, and administrative influence PM-JAY utilization in Jammu and Kashmir.

As shown in Figure 1, the mapping of study variables to the framework are as follows:

- Predisposing factors (Andersen): Education and literacy influence beneficiaries’ capacity to perceive entitlements and navigate scheme processes; informational awareness (e.g., awareness of benefits; knowledge of empanelled hospitals) shapes the ability to perceive and seek care (23, 24).
- Enabling resources (Andersen): PM-JAY enrollment represents formal financial coverage, while household resources and real-world affordability remain affected by informal payments, out-of-pocket expenses, and wage loss. These relate to the ability to pay and affordability conditions (24).
- Administrative mediation (Access/health system interface): Documentation burdens and claim processes influence beneficiaries’ ability to engage with the scheme and complete the utilization pathway (24).
- Physical accessibility (Enabling + ability to reach): Road quality, transport availability, and distance shape the ability to reach care and reflect service accessibility constraints (24).
- Contextual constraints (system/environment): Conflict exposure, terrain, and infrastructure limitations in Jammu and Kashmir operate as overarching contextual factors that can moderate or constrain each pathway, reducing the effectiveness of financial protection alone (3).

Accordingly, PM-JAY utilization is modeled as the cumulative outcome of these interacting pathways: informational readiness (ability to perceive/seek), physical reachability (ability to reach), and administrative navigability (ability to engage), with financial protection acting as a necessary but often insufficient enabling condition in fragile settings. This framework directly informs the study’s empirical strategy (barrier-wise modelling) and interpretation of findings, particularly the observed pattern where informational and physical barriers show stronger independent associations with utilization than financial barriers once other constraints are controlled for (12, 24).

## 4 Methodology

### 4.1 Study design and scope

The cross-sectional quantitative survey was used in this study to discuss the impediments to PM-JAY utilization among the beneficiaries in Jammu and Kashmir. The study was carried out in two districts namely Jammu (urban administrative center) as well as Baramulla (semi-rural/conflict-affected region) to reflect the structural and contextual variation in the Union Territory. The goal of the study was to obtain both the district level variation and the individual level barriers through item-based analysis.

### 4.2 Sampling strategy

A multi-stage stratified random sampling technique was adopted. In the first stage, districts were stratified based on settlement characteristics to reflect the urban–semi-rural divide, from which Jammu (predominantly urban) and Baramulla (semi-rural and conflict-affected) districts were selected. In the second stage, urban municipal wards were randomly selected from Jammu district, while villages were randomly selected from Baramulla district. In the final stage, households were randomly drawn from officially obtained PM-JAY beneficiary lists within the selected wards and villages. A total of 320 respondents—169 from Jammu and 151 from Baramulla—were interviewed. Respondents were eligible if they had been enrolled under PM-JAY for at least 1 year at the time of the survey. A pre-tested questionnaire was used to undertake face-to-face structured interviews in February to April 2025. Ethical approval for this study was obtained from the Institutional Ethics Committee, Government Degree College Kishtwar (Approval No.: Kst/Coll/25/IEC/25/01, dated 31 January 2025). Written informed consent was obtained from all participants prior to data collection, and confidentiality of respondents was strictly maintained throughout the study.

### 4.3 Survey instrument and reliability

Data were collected using a structured, pre-tested questionnaire administered through face-to-face interviews. All barrier-related variables were measured using a five-point Likert scale ranging from 1 (Not a barrier at all) to 5 (Very high barrier). To assess internal consistency, reliability analysis was conducted for grouped barrier constructs. The results indicate acceptable reliability, with Cronbach’s alpha values above the conventional threshold of 0.70 for all major constructs, confirming the internal consistency of the survey instrument.

### 4.4 Measurement of variables

PM-JAY utilization, the dependent variable, was measured as a binary outcome, coded as 1 if the respondent reported having used PM-JAY services and 0 otherwise.

All barrier-related variables—including financial (out-of-pocket expenses, informal payments, wage loss), administrative (documentation hurdles, claim rejections), informational (awareness of scheme benefits, knowledge of empanelled hospital lists), and physical barriers (road quality, transport availability, distance to health facility)—were measured using a five-point Likert scale, where 1 = Not a barrier at all, 2 = Low barrier, 3 = Moderate barrier, 4 = High barrier, and 5 = Very high barrier. These variables were treated as ordinal measures, with higher scores indicating greater perceived severity.

For regression analysis, Likert-scale barrier variables were treated as ordinal continuous predictors, and odds ratios were interpreted as reflecting the change in probability of PM-JAY utilization associated with a one-unit increase in perceived barrier severity.

Socio-demographic variables such as gender and district of residence were measured on a nominal scale, while education level and income categories were measured as ordinal variables. For descriptive analysis, Likert-scale items were summarized using means and standard deviations, while inferential analysis employed logistic regression to estimate adjusted odds ratios for PM-JAY utilization.

### 4.5 Analytical framework

A combination of descriptive and inferential statistical techniques was employed to address the study objectives. Descriptive statistics were used to summarize socio-demographic characteristics and to assess the perceived severity of financial, administrative, informational, and physical barriers to PM-JAY utilization. Chi-square tests of independence were applied to examine associations between categorical variables, such as district of residence, socio-demographic characteristics, awareness levels, and PM-JAY utilization outcomes, thereby addressing differences across population subgroups.

Pearson correlation analysis was conducted to explore bivariate relationships among barrier variables and to assess the potential for multicollinearity prior to multivariate modeling. Finally, a multivariate binary logistic regression model was employed to estimate the independent effects of each barrier category on PM-JAY utilization while controlling for other factors. District-level differences (Jammu vs. Baramulla) were assessed using chi-square tests of independence, while logistic regression was conducted on the pooled sample without interaction terms or stratified models. This approach allows identification of barriers that exert statistically significant independent effects on utilization, directly addressing the study’s objective of determining critical constraints to effective scheme use.

The probability of PM-JAY utilization will be modeled using binary logistic regression. The core regression model is specified as:

Proposed logistic regression model.


$$\begin{array}{l} \text{logit}[P(Y=1)]=\beta 0+\beta 1(\mathrm{OOP})+\beta 2(\mathrm{IP})+\beta 3(\mathrm{WL})\\ +\beta 4(\mathrm{DOC})+\beta 5(\mathrm{CR})+\beta 6(\mathrm{AWB})+\beta 7(\mathrm{HL})\\ +\beta 8(\mathrm{RQ})+\beta 9(\mathrm{TA})+\beta 10(\text{DIST}) \end{array}$$

Where:

β0 is the intercept; OOP = out-of-pocket expenses; IP = informal payments; WL = wage loss; DOC = documentation hurdles; CR = claim rejections; AWB = awareness of scheme benefits; HL = knowledge of empanelled hospital lists; RQ = road quality; TA = transport availability; DIST = distance to health facility.

Odds ratios represent the adjusted effect of each individual barrier item on PM-JAY utilization. They were computed to interpret the magnitude of effect for each barrier. A *p*-value threshold of 0.05 was used to determine statistical significance. All analyses were conducted using SPSS Version 26.

This approach allows for both individual-level inference and broader district-level comparisons, building on the methodological guidance provided by prior UHC research in Ethiopia and India (7).

## 5 Results

Table 1 presents the socio-demographic characteristics of the respondents from Jammu and Baramulla districts. Among the 320 enrolled PM-JAY beneficiaries, 48.4% reported having utilized PM-JAY services, while 51.6% had not utilized the scheme despite being enrolled. The sample included respondents across different age groups, education levels, income categories, and employment statuses, with representation from both urban and semi-rural settings.

**Table 1:** Socio-demographic profile and PM-JAY utilization status of respondents.

Variable	Category	Frequency (*n* = 320)	Percentage (%)
Gender	Female	145	45.3
	Male	175	54.7
District	Baramulla	151	47.2
	Jammu	169	52.8
Education	Illiterate	81	25.3
	Primary	88	27.5
	Secondary	79	24.7
	Graduate	72	22.5
Caste	General	67	20.9
	OBC	91	28.4
	SC	75	23.4
	ST	87	27.2
Utilization of PM-JAY	No (0)	165	51.6
	Yes (1)	155	48.4

Source: Primary survey.

Table 2 summarizes the descriptive statistics for perceived financial, administrative, informational, and physical barriers measured on a five-point Likert scale. Among financial barriers, informal payments recorded the highest mean score (*M* = 3.21), followed by wage loss (*M* = 3.01) and out-of-pocket expenditure (*M* = 2.97). Administrative barriers such as documentation requirements had a mean score of 2.83 health literacy and awareness gaps, including awareness of scheme benefits and knowledge of empanelled hospital lists, recorded mean values above 3.0, while physical barriers such as transport availability and road quality also showed moderate to high mean scores.

**Table 2:** Descriptive statistics for key barriers on a five-point Likert scale (*N* = 320).

Variable	Mean (*M*)	Std. Deviation (SD)	Interpretation
Out-of-pocket expenses	2.97	1.40	High barrier
Informal payments	3.21	1.35	High barrier
Wage loss	3.01	1.39	High barrier
Documentation hurdles	2.83	1.42	Access barrier
Awareness of benefits	3.06	1.40	Moderate awareness
Transport availability	3.00	1.39	Neutral barrier

Source: Primary survey.

**Figure 2:**
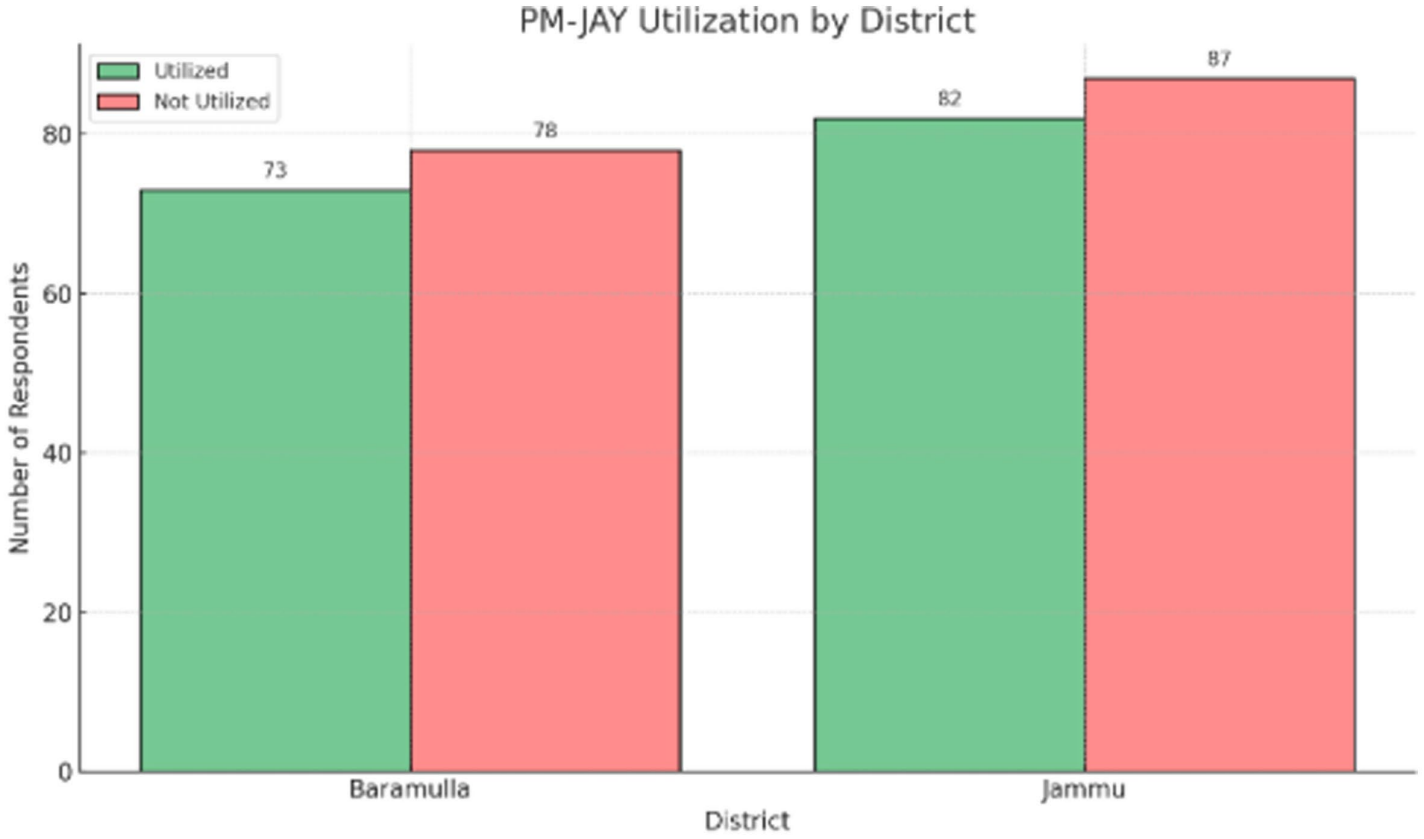
PM-JAY utilization by district. Source: Primary survey.

Figure 2 presents PM-JAY utilization across the two study districts. Utilization was reported by 48.3% of respondents in Baramulla and 48.5% of respondents in Jammu, indicating minimal variation between the two locations.

Table 3 reports the results of chi-square tests examining associations between socio-demographic variables, awareness indicators, and PM-JAY utilization. The association between district and utilization was not statistically significant (*χ*^2^ = 0.001, *p* = 0.975). Education level showed a statistically significant association with awareness of PM-JAY benefits (*p* < 0.001). Income and age group were also significantly associated with awareness levels.

**Table 3:** Chi-square test for association between district and PM-JAY utilization.

Variable	*χ*^2^	df	*p*-value
District × PMJAY utilization	0.001	1	0.975

Source: Primary survey.

Table 4 reveals the awareness of scheme benefits (*r* = 0.148, *p* < 0.01) and knowledge of empanelled hospital lists (*r* = 0.121, *p* < 0.05) were positively correlated with PM-JAY utilization. Financial and administrative barrier variables showed weak and statistically non-significant correlations with utilization. Correlations among predictor variables were low to moderate, indicating no serious multicollinearity concerns.

**Table 4:** Pearson correlation matrix among PM-JAY utilization and barrier variables.

Variables	1	2	3	4	5	6	7	8	9	10	11
1. Utilization PMJAY	–										
2. OOP expenses	0.022	–									
3. Informal payments	0.026	−0.083	–								
4. Wage loss	−0.022	−0.142 (0.011)	0.018	–							
5. Claim rejections	−0.028	0.085	−0.083	0.001	–						
6. Documentation hurdles	0.016	−0.009	0.039	0.036	0.037	–					
7. Awareness of benefits	0.148 (0.008)	0.014	0.012	0.026	0.068	0.000	–				
8. Hospital lists	0.121 (0.031)	0.029	−0.145 (0.009)	0.038	0.069	0.065	−0.003	–			
9. Distance	−0.039	−0.029	−0.027	0.003	0.005	−0.052	−0.053	−0.058	–		
10. Road quality	−0.090	−0.015	0.009	−0.021	0.029	−0.046	0.013	−0.063	0.040	–	
11. Transport availability	0.068	0.003	0.052	0.020	0.118 (0.036)	−0.090 (0.090)	0.024	−0.045	−0.113 (0.036)	−0.076	–

Source: Primary survey.

Only the lower-triangular portion of the Pearson correlation matrix is reported to avoid redundancy. Diagonal elements represent self-correlations, and dashes indicate values that are not displayed by design rather than missing data.

The correlation analysis helps to understand the relationships between PM-JAY utilization and all important variables of barriers. Of the independent variables, health literacy and awareness gaps indicated the best associations with utilization which were positive. In particular, the awareness schemes benefits (*r* = 0.148) and awareness of empanelled hospitals lists (*r* = 0.121) was found to be positively associated with service use, which means that more informed beneficiaries will use PM-JAY more than the less informed beneficiaries. Conversely, financial hindrances like out-of-the-pocket (*r* = 0.022), payments in kind (*r* = 0.026), and loss of wages (*r* = −0.022) had insignificant relationships with utilization. Very weak associations were also observed concerning administrative barriers such as documentation (*r* = 0.016) and claims denied (*r* = −0.028), indicating that these factors have minor direct effects in the utilization decision in the current dataset. Even though physical access variables produced only small effects in the right direction, they were stronger. Utilization was found to be negatively correlated with the level of the capacity of the roads (*r* = −0.090) and with distance to health facilities (*r* = −0.039) which shows that the poor infrastructure could also be a deterrent to the use of the scheme though the impact is negligible. A limited positive association was indicated by transport availability (*r* = 0.068), implicating that the facility of access through ease of transport could help.

Table 5 presents chi-square test results examining associations between selected socio-demographic characteristics, awareness indicators, and PM-JAY-related outcomes. Significant associations were observed between age, education, income, and district with awareness of PM-JAY benefits (*p* < 0.01). Gender and employment status were significantly associated with satisfaction with services, while hospital visit frequency was significantly related to perceived scheme benefits.

**Table 5:** Chi-square test results for selected variables under PM-JAY.

Variables tested	*χ*^2^ value	df	*p*-value
Age group × Awareness of PM-JAY	21.763	4	0.0002
Education level × Awareness of PM-JAY	35.214	6	0.0001
District × Awareness of PM-JAY	42.187	5	0.0000
Income level × Awareness of PM-JAY	18.092	3	0.0009
Gender × Satisfaction with services	12.038	1	0.0005
Employment status × Satisfaction with services	26.118	4	0.0000
Type of hospital × Experience with PM-JAY	33.506	3	0.0000
Awareness source × Enrollment status	22.779	3	0.0002
Hospital visit frequency × Scheme benefits	28.369	2	0.0000

Source: Primary survey.

Table 6 presents the results of the multivariate binary logistic regression model estimating adjusted odds ratios for PM-JAY utilization. Informational variables, including awareness of scheme benefits (OR = 1.253, *p* < 0.05) and availability of hospital lists (OR = 1.245, *p* < 0.05), were statistically significant predictors of utilization. Physical accessibility, measured through road quality (OR = 0.761, *p* < 0.05), was also a significant determinant. Out-of-pocket expenses, informal payments, and wage loss were not statistically significant predictors of PM-JAY utilization.

**Table 6:** Logistic regression results for PM-JAY utilization (Barrier-based model).

Barrier Category	Predictor	*B*	SE	Wald	*p*	OR (Exp(B))
Administrative barriers	Documentation hurdles	0.085	0.083	1.043	0.307	1.089
	Claim rejections	−0.08	0.083	0.934	0.334	0.923
Financial barriers	Informal payments	0.072	0.092	0.61	0.437	1.075
	Out-of-pocket expenses	0.044	0.087	0.253	0.615	1.045
	Wage loss due to treatment	−0.04	0.087	0.213	0.645	0.961
Health literacy and awareness gaps	Availability of hospital lists	0.219	0.099	4.871	0.027	1.245
	Awareness of benefits	0.226	0.111	4.124	0.042	1.253
Physical barriers	Road quality	−0.274	0.128	4.571	0.033	0.761
	Transport availability	0.098	0.088	1.238	0.133	1.103
	Distance to health facility	−0.011	0.061	0.017	0.896	0.989

Source: Primary survey.

**Figure 3:**
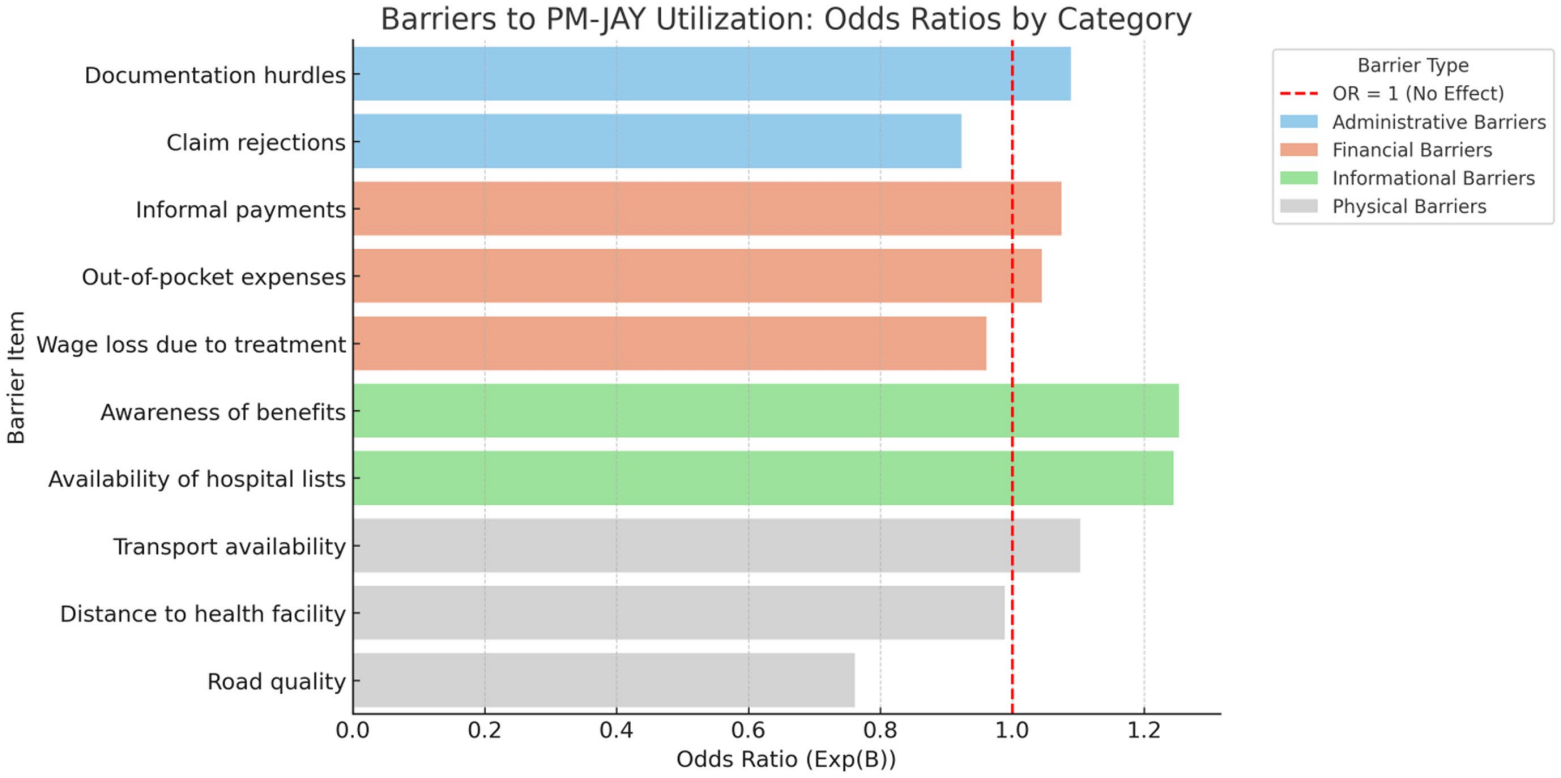
Barriers to PM-JAY utilization: odds ratio by category. Source: Primary survey.

As shown in Figure 3, the logistic regression model identified the influence of financial, administrative, informational, and physical barrier to the probability of using the PM-JAY by the beneficiaries. Among the 10 predictors that were used in the model, three were found to be statistically significant at 5 percent level. In the case of health literacy and awareness gaps, a positive and significant impact on utilization was determined by awareness of scheme benefits (*B* = 0.226, *p* = 0.042, OR = 1.253) and the availability of hospital lists (*B* = 0.219, *p* = 0.027, OR = 1.245). This is indicative of more information-informed beneficiaries having a high probability of accessing PM-JAY services. Awareness of scheme benefits was positively associated with PM-JAY utilization (OR = 1.253, *p* = 0.042), as was awareness of empanelled hospital lists (OR = 1.245, *p* < 0.05). Road quality showed a statistically significant negative association with utilization (OR = 0.761, *p* = 0.033). Financial and administrative barriers did not demonstrate statistically significant associations when other variables were controlled. On the other hand, the other variables, in particular those that are concerned with the financial barriers that encompass out-of-pocket payment and wage loss, and informal payments did not provide statistically significant results, even though in the descriptive analysis they were ranked higher than the other variables. In a similar manner, administrative barriers, such as documentation barriers and claim denials, were not substantial predictors in the model implying that, although such barriers can have large prevalence, they are not independent predictors of utilization when other factors are held constant.

## 6 Discussion

The study results provide valuable information in solving the complicated relationship between individual-level barriers and utilization of PM-JAY in Jammu and Kashmir, using Jammu and Baramulla districts as a case study. Even though the scheme was aimed at ensuring financial security for economically vulnerable groups through cashless hospitalization, the results show that not more than half (48.4%) of enrolled beneficiaries actually utilized PM-JAY services. This low uptake indicates that official enrollment alone is not a sufficient determinant of meaningful access. The conceptual framework proposed in this study provides a coherent lens through which these findings can be interpreted. Consistent with the adapted Andersen–Levesque framework, PM-JAY utilization in Jammu and Kashmir emerges not as a direct function of financial coverage alone, but as the outcome of interacting predisposing, enabling, and mediating factors. The framework anticipated that informational readiness (ability to perceive and seek care) and physical accessibility (ability to reach care) would play a decisive role in translating scheme eligibility into actual utilization, particularly in a fragile and geographically constrained setting. The empirical results strongly align with this expectation, as informational variables and road quality retained statistical significance in the regression model, while financial and administrative barriers—though prevalent—did not independently predict utilization once other constraints were controlled for.

The chi-square analysis indicates that district location has a negligible influence on PM-JAY utilization (*p* = 0.975), suggesting that the observed barriers are not district-specific but systemic and widespread across both urban and semi-rural settings. This finding corresponds with the study by Khan et al. (26), who observed that distress financing and out-of-pocket spending persisted across different tertiary care centers under Ayushman Bharat. The absence of geographic variation implies that access restrictions are not confined to specific locations but are embedded within broader systemic constraints. Consequently, the results indicate the need for universal administrative and informational reforms rather than narrowly localized interventions.

Descriptive statistics reveal high levels of financial barriers, including out-of-pocket expenditure, wage loss, and informal payments. Nevertheless, the logistic regression outcomes indicate that these financial constraints do not emerge as independent predictors of utilization once other factors are held constant. The divergence between descriptive prominence and regression-based significance reflects a methodological distinction rather than an empirical contradiction. While descriptive statistics capture perceived severity and frequency, regression analysis isolates the independent contribution of each factor. In this context, financial barriers appear to function as background conditions that are widely experienced by beneficiaries, whereas informational and infrastructural constraints exert a more direct influence on PM-JAY utilization behavior. This divergence does not imply that financial hardship is unimportant; rather, it suggests that its influence on utilization is indirect and mediated through informational and infrastructural pathways. This interpretation is supported by Rasool and Geer (27), who found that despite the prevalence of financial burden under PM-JAY in Jammu and Kashmir, beneficiaries most frequently cited lack of awareness and procedural complexity as reasons for non-utilization.

Health literacy and awareness gaps identified in the regression model are of particular importance, as awareness of scheme entitlement and knowledge of empanelled hospital lists significantly increased the odds of utilization. These findings align with the systematic review by Reshmi et al. (28), which highlighted the strong influence of awareness campaigns and clarity regarding entitlements on health scheme utilization across India.

In the current study, increased awareness raised the likelihood of utilization by more than a quarter, underscoring the urgency of strengthening Information, Education, and Communication (IEC) activities, especially among semi-literate and illiterate populations. Given the strong association between education level and scheme awareness observed in the chi-square analysis (*p* < 0.001), IEC strategies should prioritize non-written and interpersonal modes of communication. These may include radio and local broadcast messaging, community meetings facilitated by ASHA and Anganwadi workers, engagement of local panchayat and religious leaders, and verbal counseling at health facilities. Such approaches are more likely to reach illiterate and primary-educated populations for whom text-heavy information campaigns may be ineffective. Furthermore, limited awareness of empanelled hospital lists reflects health system navigation support mechanisms deficiencies that could be addressed through enhanced digital tools and frontline outreach, as suggested by Shende and Wagh (6).

Physical accessibility also emerged as one of the most important determinants of PM-JAY utilization, with poor road quality significantly reducing the likelihood of scheme use (OR = 0.761). This effect is particularly pronounced in a region such as Jammu and Kashmir, which is characterized by mountainous terrain, conflict exposure, and underdeveloped transport and road infrastructure. Bajpai and Wadhwa (29) similarly observed that inadequate rural infrastructure restricts the reach of Health and Wellness Centres (HWCs), thereby undermining the broader objectives of Ayushman Bharat. The present findings affirm this relationship in the context of hospital-based care and underscore the necessity of sustained investment in health-related infrastructure, especially in regions where geographic constraints pose substantial barriers to access.

Interestingly, administrative obstacles such as documentation challenges and claim denials—although rated as moderate barriers in the descriptive analysis—did not emerge as statistically significant predictors of utilization in the regression model. This suggests that these barriers may exert a more secondary or background influence and may only become consequential when combined with other constraints. Sarwal and Kumar (11) note that a plurality of beneficiaries disengage from health insurance systems due to cumulative fatigue, distrust, and procedural burden.

Furthermore, chi-square analysis reveals significant associations between awareness levels and socio-demographic characteristics such as education, income, and age. This pattern indicates that social determinants of health continue to shape individuals’ capacity to utilize PM-JAY services, consistent with evidence from other country contexts (30) and national-level studies in India (4). The present findings suggest that health literacy—closely linked to education—remains one of the most robust pathways toward equitable scheme utilization. In this regard, community health facilitation, ASHA training, and village-level awareness programs can play a pivotal role in improving uptake.

The conflict-affected nature of Jammu and Kashmir further intensifies these barriers and provides important context for interpreting the study findings. Prolonged conflict has contributed to chronic underinvestment in transport and health infrastructure, recurrent mobility restrictions, and disruptions in service delivery, all of which constrain beneficiaries’ ability to physically reach empanelled facilities (3). Conflict also weakens institutional trust and complicates administrative engagement, as populations exposed to prolonged insecurity may be less likely to navigate formal procedures or rely on state-led schemes (31). In addition, periodic shutdowns, displacement, and communication disruptions undermine the effectiveness of information dissemination, exacerbating awareness gaps even among enrolled beneficiaries. These dynamics suggest that conflict does not operate as an isolated barrier, but rather amplifies informational, physical, and administrative constraints, thereby limiting the extent to which financial protection alone can translate into effective healthcare utilization.

The study also contributes to the growing body of literature examining public trust and engagement with health insurance systems in fragile settings. Vitsupakorn et al. (31) report substantial regional variation in early PM-JAY implementation, driven by differences in institutional capacity and outreach effectiveness. This insight is particularly relevant for Jammu and Kashmir, where conflict-related mobility restrictions and administrative fragility often limit formal citizen engagement with public schemes. Overall, the evidence supports the conclusion that informational and physical access barriers are more central in shaping PM-JAY utilization than financial or administrative constraints alone. Policy interventions should therefore move beyond enrollment expansion and prioritize enabling conditions such as transport infrastructure, scheme awareness, hospital mapping, and beneficiary health system navigation support. These findings also highlight the value of granular, barrier-specific statistical models for informing policy, rather than relying on aggregate service utilization indicators.

## 7 Conclusion

This paper highlights the significant sophistication of all obstacles that affect the use of PM-JAY services at both Jammu and Baramulla districts in Jammu and Kashmir. Although they were enrolled in one of the biggest publicly funded health assurance schemes in the world, less than 50 percent of the survey participants said that they utilized its services which clearly shows the gap between enrollment and utilization. Whereas the financial and administrative barriers are some of the most frequently experienced barriers that are discussed, logistic regression analysis shows that informational and physical barriers have a more deterministic impact on the service uptake. In particular, the awareness of scheme benefits and knowledge of empanelled hospitals also significantly raised the probability of usage whereas bad roads negatively affected it considerably. These results highlight two important issues regarding structural and informational accessibility as the prime determinants in enhancing health outcomes in fragile environments rather than financial protection alone. Notably, the research does not detect significant difference in usage between the urban district of Jammu and the semi-rural/conflict-affected district of Baramulla, which indicate that such a barrier can be viewed as systemic rather than confined to the local topic. Moreover, chi-square tests show that education, income, and age are crucial factors that combine with the level of awareness implying the imbalance in health literacy. In order to shift toward real universal health coverage in the areas located in Jammu and Cashmir, policymakers should designate a focus on the IEC campaigns, enhanced last-mile connectivity, hospital mapping process, and frontline wayfinding assistance. It is not enough to increase coverage on paper, but the priority should be granted to facilitating real access particularly to disadvantaged and geographically isolated populations. The present study adds to the evidence base that demands barrier- sensitive and context-specific policy changes in the area of public health insurance based on the empirical rigor and ground realities.

## Data Availability

The original contributions presented in the study are included in the article/supplementary material, further inquiries can be directed to the corresponding author.
